# Cushing syndrome caused by an ectopic ACTH-producing pituitary adenoma of the clivus region: A rare case report and literature review

**DOI:** 10.1097/MD.0000000000034137

**Published:** 2023-06-23

**Authors:** Yutao He, Ziyi Tang, Na Tang, Yu Lu, Fangfang Niu, Jiao Ye, Zheng Zhang, Chenghong Fang, Lei Yao

**Affiliations:** a Department of Laboratory Medicine, Guiqian International General Hospital, Guiyang, Guizhou Province, China.

**Keywords:** clivus region, Cushing, Ectopic ACTH, like appearance, producing pituitary adenoma

## Abstract

Ectopic ACTH-producing pituitary adenoma (EAPA) of the clivus region is extraordinarily infrequent condition and merely a few reports have been reported to date.

**Patient concerns:**

The patient was a 53-year-old woman who presented with Cushing-like appearances and a soft tissue mass in the clivus region.

**Diagnoses:**

The final diagnosis of clivus region EAPA was established by clinical, radiological and histopathological findings.

**Interventions:**

The patient underwent gross total clivus tumor resection via transsphenoidal endoscopy.

**Outcomes:**

Half a year after surgery, the patient Cushing-like clinical manifestations improved significantly, and urinary free cortisol and serum adrenocorticotropin (ACTH) returned to normal.

**Lessons:**

Given the extreme scarcity of these tumors and their unique clinical presentations, it may be possible to misdiagnose and delayed treatment. Accordingly, it is especially crucial to summarize such lesions through our present case and review the literature for their precise diagnosis and the selection of optimal treatment strategies.

## 1. Introduction

Pituitary adenoma arises from the anterior pituitary cells and is the commonest tumor of the sellar region.^[1]^ It makes up approximately 10% to 15% of all intracranial tumors.^[2]^ Ectopic pituitary adenoma (EPA) is defined as a pituitary adenoma that occurs outside the sellar area and has no direct connection to normal pituitary tissue.^[3]^ The most frequent sites of EPA are the sphenoid sinus and suprasellar region, and much less frequent sites including the clivus region, cavernous sinus, and nasopharynx.^[4]^

Hypercortisolism and the series of symptoms it leads to is termed Cushing syndrome (CS).^[5]^ CS is classified into adrenocorticotropin (ACTH)-dependent and ACTH-independent CS depending on the cause, accounting for 80% to 85% and 15% to 20% of cases, respectively.^[6]^ Pituitary adenoma accounts for ACTH-dependent CS 75% to 80%, while ectopic ACTH secretion accounts for the remaining 15% to 20%.^[7]^ Ectopic CS is a very rare disorder of CS caused by an ACTH-secreting tumor outside the pituitary or adrenal gland.^[8]^ It has been reported that ectopic ACTH-producing pituitary adenoma (EAPA) can occur in the sphenoid sinus, cavernous sinus, clivus, and suprasellar region,^[9]^ with EAPA in the clivus region being extremely rare, and merely 6 cases have been reported in the English literature (Table 1).^[10–15]^ Furthermore, as summarized in the Table 1, EAPA in the clivus area has unique symptoms, which may lead to misdiagnosis as well as delay in treatment. Therefore, we herein described a case of CS from an EAPA of the clivus region and reviewed relevant literature for the purpose of further understanding this extraordinarily unusual condition.

**Table 1:** Literature review of cases of primary clival ectopic ACTH-producing pituitary adenoma (including the current case).

Reference	Age (yr)/sex	Symptoms	Imaging findings	Maximum tumor diameter (mm)	Preoperative elevated hormone	IHC	Surgery	RT	Follow-up (mo)	Outcome
Ortiz et al 1975^[10]^	15/F	NA	NA	NA	NA	NA	Right transfrontal craniotomy, NA	Yes	NA	Symptomatic relief
Anand et al 1993^[11]^	58/F	Anosphrasia, blurred vision, occasional left frontal headache,	Routine radiographic evaluation revealed a clival tumor and nasopharyngeal mass with bone erosion. MRI demonstrated a Midline homogeneous mass.	30	ACTH	ACTH in a few isolated cells	Maxillotomy approach, GTR	Yes	12	Symptomatic relief
Pluta et al 1999^[12]^	20/F	Cushing syndrome	MRI revealed a hypodense contrast-enhancing lesion.	NA	ACTH	ACTH	Transsphenoidal surgery, GTR	No	18	Symptomatic relief
Shah et al 2011^[13]^	64/M	Facial paresthesias, myalgias, decreased muscle strength, and fatigue	CT imaging showed a clival mass.	21	ACTH	ACTH	NA, GTR	No	7	Symptomatic relief
Aftab et al 2021^[14]^	62/F	Transient unilateral visual loss	MRI showed a T2 heterogeneously enhancing hyperintense lesion.	21	No	ACTH	Transsphenoidal resection, GTR	NO	6	Symptomatic relief
Li et al 2023^[15]^	47/F	Bloody nasal discharge, dizziness and headache	CT revealed an ill-defined mass eroding the adjacent bone. MRI T1 showed a heterogeneous mass with hypointensity, hyperintensity on T2-weighted images and isointensity on diffusion-weighted images.	58	NA	ACTH	Transsphenoidal endoscopy, STR	Yes	2	Symptomatic relief
Current case	53/F	Headache, and dizziness, Cushing syndrome	CT demonstrated bone destruction and a soft tissue mass. MRI T1 revealed irregular isointense signal, and MRI T2 showed isointense signal/slightly high signal.	46	ACTH	ACTH	Transsphenoidal endoscopy, GTR	NO	6	Symptomatic relief

ACTH = adrenocorticotropin, CT = computed tomography, GTR = gross total resection, IHC = immunohistochemistry, MRI = magnetic resonance imaging, NA = not available, RT = radiotherapy, STR = subtotal resection.

## 2. Case presentation

A 53-year-old female presented to endocrinology clinic of our hospital with headache and dizziness for 2 years and aggravated for 1 week. Her past medical history was hypertension, with blood pressure as high as 180/100 mm Hg. Her antihypertensive medications included amlodipine besylate, benazepril hydrochloride, and metoprolol tartrate, and she felt her blood pressure was well controlled. In addition, she suffered a fracture of the thoracic vertebrae 3 month ago; and bilateral rib fractures 1 month ago. Physical examination revealed that the patient presented classical Cushing-like appearances, including moon face and supraclavicular and back fat pads, and centripetal obesity (body mass index, 25.54 kg/m^2^) with hypertension (blood pressure, 160/85 mm Hg).

Laboratory studies revealed high urinary free cortisol levels at 962.16 µg/24 hours (reference range, 50–437 µg/24 hours) and absence of circadian cortisol rhythm (F _[0_ _am__]_ 33.14 µg/dL, F _[8_ _am__]_ 33.52 µg/dL, F _[4_ _pm__]_ 33.3 µg/dL). ACTH levels were elevated at 90.8 pg/mL (reference range, <46 pg/mL). The patient low-dose dexamethasone suppression test demonstrated the existence of endogenous hypercortisolism. High-dose dexamethasone suppression test results revealed that serum cortisol levels were suppressed by <50%, suggesting the possibility of ectopic ACTH-dependent CS. Serum luteinizing hormone and serum follicle stimulating hormone were at low levels, <0.07 IU/L (reference range, 15.9–54.0 IU/L) and 2.57 IU/L (reference range, 23.0–116.3 IU/L), respectively. Insulin-like growth factor-1, growth hormone (GH), prolactin (PRL), thyroid stimulating hormone, testosterone, progesterone and estradiol test results are all normal. Oral glucose tolerance test showed fasting glucose of 6.3 mmol/L and 2-hour glucose of 18.72 mmol/L; glycosylated hemoglobin (HbA1c) was 7.1%. Serum potassium fluctuated in the range of 3.14 to 3.38 mmol/L (reference range, 3.5–5.5 mmol/L), indicating mild hypokalemia.

**Figure 1.**
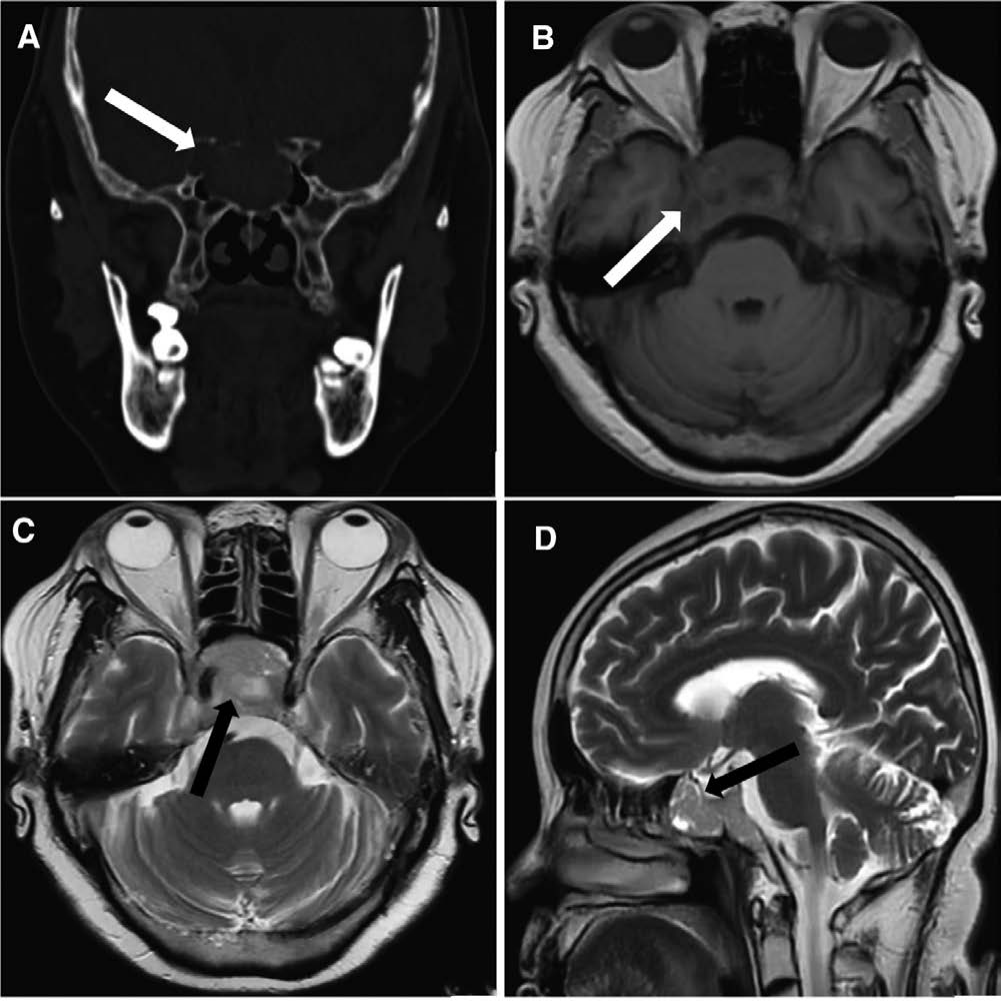
Radiological findings. (A) CT demonstrated bone destruction and a soft tissue mass on the occipital clivus (white arrow). (B) Axial view of the MR T1 revealed irregular isointense signal in the sphenoid sinus and saddle area (white arrow). (C and D) Axial view and sagittal view of the MR T2 showed isointense signal/slightly high signal in the sphenoid sinus and saddle area (black arrow). CT = computed tomography.

High-resolution computed tomography (CT) scan of the sinuses revealed osteolytic bone destruction of the occipital clivus and a soft tissue mass measuring 20 mm × 30 mm × 46 mm (Fig. 1A). The mass filled the bilateral sphenoid sinuses and involved the cavernous sinuses, but the pituitary was normal. Cranial MR scan showed the T1W1 isointense signal and the T2W1 isointense signal/slightly high signal in the sphenoid sinus and saddle area (Fig. 1B–D). Bone density test indicated osteoporosis.

**Figure 2.**
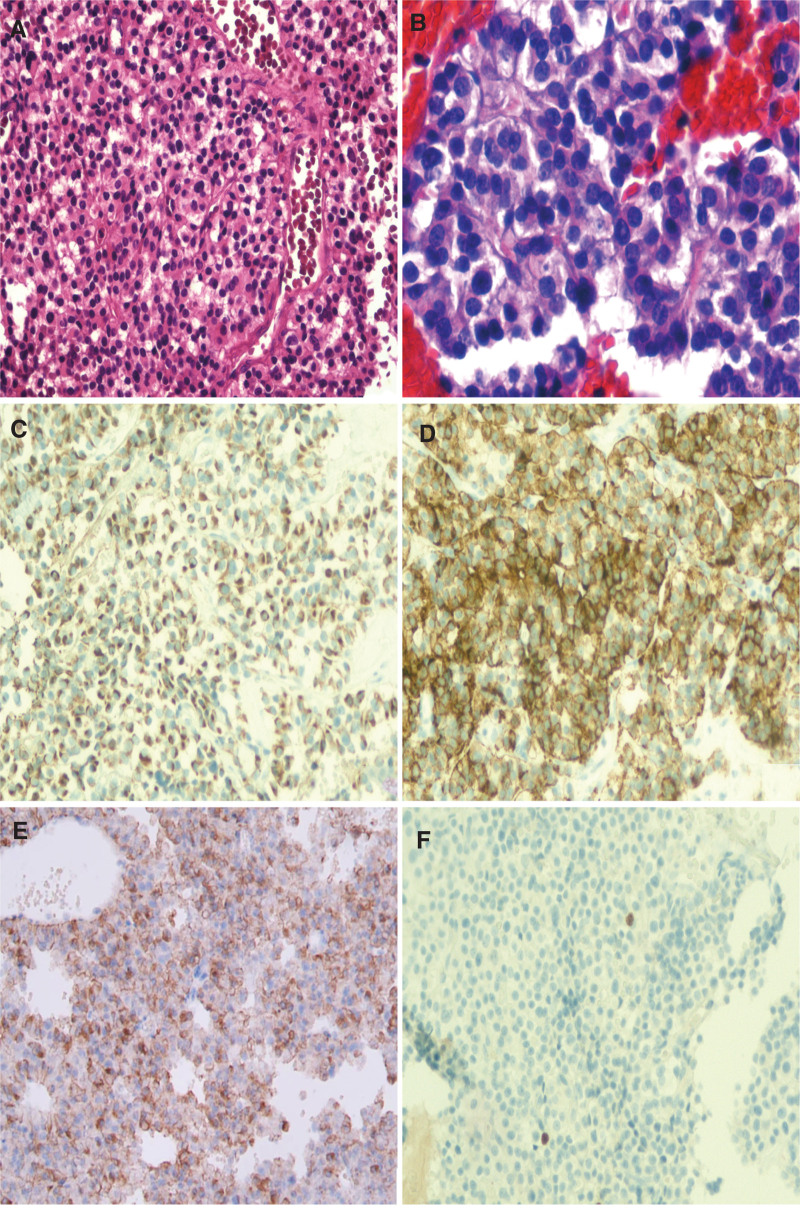
HE and immunohistochemical findings. (A) Histologic sections revealed morphologically homogeneous tumor cells in nests with a prominent and delicate vascularized stroma (H&E, × 200). (B) The tumor cells had fine chromatin with visible nuclei and rare mitoses (H&E, × 400). CK (C), CgA (D) and ACTH (E) immunohistochemically showed diffuse reactivity of the tumor cells (SP × 200). (F) The proliferation index is <1% on Ki-67 staining (SP × 200).

Subsequently, the patient underwent gross total clivus tumor resection via transsphenoidal endoscopy. During surgery, the tumor was found to be light red in color with a medium texture, and the tumor tissue protruded into the sphenoidal sinus cavity and eroded the clival area. Histologically, the tumor cells were nested, with interstitially rich blood sinuses and organoid arrangement (Fig. 2A). The tumor cells were relatively uniform in size, with light red cytoplasm, delicate pepper salt-like chromatin, and visible nucleoli (Fig. 2B). In addition, mitosis of tumor cells was extremely rare. Immunohistochemically, the neoplasm cells were diffuse positive for CK (Fig. 2C), CgA (Fig. 2D), ACTH (Fig. 2E), Syn and CAM5.2, with low Ki-67 labeling index (<1%) (Fig. 2F). Simultaneously, all other pituitary hormone markers like GH, thyroid stimulating hormone, PRL, luteinizing hormone, as well as follicle stimulating hormone were negatively expressed. On the basis of these medically historical, clinical, laboratorial, morphologic, and immunohistochemical findings, the final pathological diagnosis of an EAPA was established.

When evaluated 2 months after surgery, her Cushing-like characteristics had well improved, and her blood pressure was normal. Furthermore, her serum cortisol and ACTH returned to the normal levels. Six-month postoperative follow-up revealed that serum cortisol and ACTH were stable at normal levels, and no signs of tumor recurrence were detected on imaging.

## 3. Discussion

EAPA is defined as an ACTH-secreting ectopic adenoma located outside the ventricles, and has no continuity with the normal intrasellar pituitary gland.^[9]^ ACTH promotes cortisol secretion by stimulating the adrenal cortical fasciculus. The clinical manifestations of hypercortisolism are diverse, and the severity is partly related to the duration of the cortisol increase.^[8]^ Clival tumors are typically uncommon, accounting for 1% of all intracranial tumors. There are many differential diagnoses for clival lesions, including the most common chordoma (40%), meningioma, chondrosarcoma, astrocytoma, craniopharyngioma, germ cell tumors, non-Hodgkin lymphoma, melanoma, metastatic carcinoma, and rarely pituitary adenoma.^[16]^ The commonest clival EPA is a PRL adenoma, followed by null cell adenoma, and the least common are ACTH adenoma and GH adenoma.^[2]^ The clival EAPA is extremely unwonted, and only 6 other cases apart from ours have been reported in literature so far (Table 1).

The average age of the patients with these tumors was 48 years (range, 15–64 years). There was a obvious female predominance with a female-to-male prevalence ratio of 6:1. Only 2 patients (2/6, 33.3%) with reported clinical symptoms, including our patients, presented with overt clinical manifestations of CS. Compression of the mass on adjacent structures (e.g., nerves) may result in anosphrasia, visual impairment, headache, myalgias, decreased muscle strength, dizziness and facial sensory abnormalities. The diagnosis and localization of these tumors relied heavily on radiological imaging. Head MRI was the most basic method used for them detection, for localization adenomas and their invasion of surrounding structures to guide the choice of treatment and surgical options methods. Radiographic characteristics had been reported in 6 patients with EAPA in the clivus region. All of these patients (6/6, 100%) had initial positive findings of sellar MRI (or CT) identifying an ectopic adenoma before surgery. MR T1 was usually a low-intensity or isointense signal, while MR T2 was usually an isointense or slightly higher signal. The maximum diameter of the tumor was reported in 5 cases, with the mean maximum diameter was 35.2 mm (range, 21–55 mm) according to preoperative MRI and intraoperative observations. As summarized in Table 1, 4/5 clival EAPA cases secreted ACTH. Histologically, all cases (6/6, 100%) expressed ACTH scatteredly or diffusely.

The gold standard for the treatment of CS caused by EAPA was the surgical removal of EPA, which was essential to achieve remission and histological confirmation of the disease.^[9]^ The most common method of EAPA resection in the clivus region was transsphenoidal sinus resection (4/6, 66.67%), followed by craniotomy (1/6, 16.67%) and maxillary osteotomy (1/6, 16.67%). Transsphenoidal endoscopic surgery allowed resection of the EAPA and manipulation of neurovascular structures and avoidance of cerebral atrophy, whereas craniotomy allowed full exposure of the suprasellar region, direct visualization or manipulation of the adenoma, and reduced the risk of postoperative CSF leak.^[9]^ Both approaches had their advantages, and there was no consensus on which surgical approach was best for the treatment of EAPA in the slope area.^[9]^ The choice of the best surgical approach was believed to be based on the condition of the adenoma, as well as the general condition of the patient and the experience of the surgeon.^[9]^ As summarized in Table 1, most complete tumor resections were achieved regardless of the method chosen. A minority of patients underwent postoperative radiotherapy (3/7, 42.86%), and most of them had invasion of the surrounding bone tissue. All patients experienced effective postoperative relief of symptoms.

In summary, due to the rarity of this disorder, an accurate preoperative diagnosis of EAPA in the slope area is extremely challenging for the clinician or radiologist. The final precise diagnosis relies on a combination of clinical symptoms, imaging findings, histology and immunohistochemical markers. For this type of tumor, surgery is an effective treatment to relieve the clinical manifestations caused by tumor compression or hormonal secretion. The choice of postoperative adjuvant radiotherapy is mainly based on the presence of invasion of the surrounding bone tissue. Further cases may be necessary to summarize the clinical features of such lesions and to develop optimal treatment strategies.

## Acknowledgments

We would like to thank the patient and her family.

## Author contributions

**Conceptualization:** Yutao He.

**Data curation:** Ziyi Tang.

**Formal analysis:** Na Tang.

**Methodology:** Yu Lu, Fangfang Niu, Jiao Ye, Zheng Zhang, Chenghong Fang.

**Writing – original draft:** Yutao He.

**Writing – review & editing:** Yutao He, Lei Yao.
